# The blood-brain barrier in aging and neurodegeneration

**DOI:** 10.1038/s41380-022-01511-z

**Published:** 2022-03-31

**Authors:** Emily G. Knox, Maria R. Aburto, Gerard Clarke, John F. Cryan, Caitriona M. O’Driscoll

**Affiliations:** 1 Pharmacodelivery Group, School of Pharmacy, University College Cork, Cork, Ireland; 2 APC Microbiome Ireland, University College Cork, Cork, Ireland; 3 Department of Anatomy and Neuroscience, University College Cork, Cork, Ireland; 4 Department of Psychiatry and Neurobehavioural Science, University College Cork, Cork, Ireland

**Keywords:** Neuroscience, Drug discovery

## Abstract

The blood-brain barrier (BBB) is vital for maintaining brain homeostasis by enabling an exquisite control of exchange of compounds between the blood and the brain parenchyma. Moreover, the BBB prevents unwanted toxins and pathogens from entering the brain. This barrier, however, breaks down with age and further disruption is a hallmark of many age-related disorders. Several drugs have been explored, thus far, to protect or restore BBB function. With the recent connection between the BBB and gut microbiota, microbial-derived metabolites have been explored for their capabilities to protect and restore BBB physiology. This review, will focus on the vital components that make up the BBB, dissect levels of disruption of the barrier, and discuss current drugs and therapeutics that maintain barrier integrity and the recent discoveries of effects microbial-derived metabolites have on BBB physiology.

## The blood-brain barrier

The blood-brain barrier (BBB) is a highly selective interface between the blood and the brain that plays an essential role in maintaining an optimal environment for central nervous system (CNS) function and homeostasis. Without the BBB, the CNS is at risk of invasion of toxins, pathogens, immune cells, or ion dysregulation, which would lead to neuronal dysfunction and degeneration [1]. BBB function emerges from an association of brain cells, including brain endothelial cells, mural cells (pericytes and vascular smooth muscle cells), astrocytes, neurons, microglia, and a basement membrane, which is referred to as the neurovascular unit (NVU) [2]. A healthy, functional BBB implies all these components are interacting correctly. Complex tight junctions between the brain endothelial cells seal the paracellular space forming a continuous barrier, while the astrocytes, pericytes, and basement membrane surround the endothelial cells [3]. The endothelial cells are coated in glycocalyx on the luminal side and surrounded in the basement membrane on the abluminal side [4]. The basement membrane is composed of both an inner vascular basement membrane, which is secreted by endothelial cells and pericytes and an outer parenchymal basement membrane, which is secreted by astrocytes [1]. Moreover, specific transporter proteins located on the endothelial cells regulate molecules entering and exiting the brain.

An intact BBB has very low paracellular permeability and high trans-endothelial electrical resistance (TEER). Importantly, brain endothelial cells present very low rates of vesicle trafficking, limiting the transcytosis transport further contributing to a functional BBB [5].

Although not the focus of this review, it is noteworthy to mention the existence of other blood-brain/cerebrospinal fluid barriers that are essential for brain homeostasis. The meninges (dura mater, arachnoid mater, and pia mater) comprise the outermost protection of the brain. Moreover, the brain ventricles contain highly vascularized structures, the choroid plexus, composed of fenestrated blood vessels and epithelia sealed by tight junctions. This choroid plexus epithelium comprises the so-called blood-cerebrospinal fluid barrier, which establishes a barrier between the blood and the cerebrospinal fluid barrier. These barriers have been extensively reviewed elsewhere [6–8].

This review will focus on recent findings describing how different pathological states compromise BBB integrity, discuss current potential therapeutic approaches that have been explored for improving BBB integrity and slowing neurodegenerative disease pathologies, and the recent findings in microbial mediated modulation of the BBB. Firstly, it is important to introduce the structure of the BBB to understand how each aspect of normal function can be altered or manipulated, affecting the integrity of the entire system. By putting everything into context by describing the BBB structure, emphasizing how the barrier alters in age and disease, and the current drugs explored to slow the negative outcomes of age and disease through acting directly on the BBB, the goal is to exploit the easily accessible and manipulable microbiota as a potential target to modulate the BBB.

## BBB structure

### Brain endothelial cells and tight junctions

**Fig. 1:**
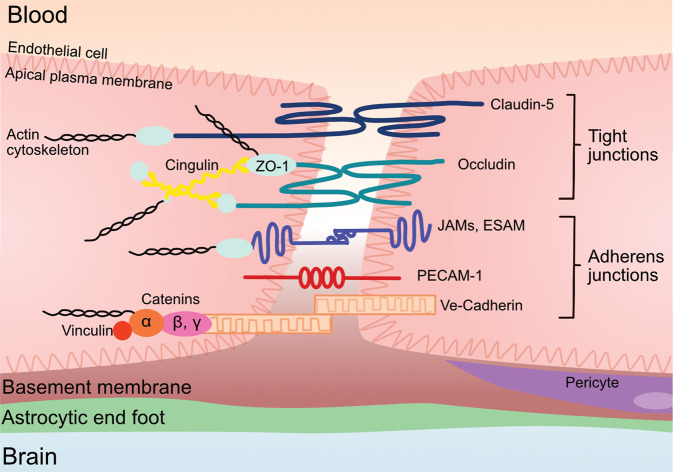
Endothelial cell tight junctions and adherens junction proteins. The tight junction proteins include claudin-5, occludin, and zonula occludins (ZO-1,2,3). Claudin-5 and occludin are both transmembreane proteins while the zonula occludens are intracellular proteins. The adherens junctions include transcellular components, JAMs, ESAM, PECAM-1, and Ve-cadeherin. The cytoplasmic catenins form a complex with Ve-cadeherin. Actin cytoskeleton helps to anchor the junctional proteins in endothelial cells.

The permeability of the BBB is one of the leading metrics used to assess BBB integrity and, it is a measure of the degree of both paracellular and transcellular transport [9]. Tight junction proteins between brain endothelial cells greatly restrict paracellular transport. Therefore, the expression and function of the tight junction proteins is often used as a metric of BBB integrity [10, 11]. Brain endothelial tight junction proteins include occludin, claudins (claudin-1, −3, −5, −12), and the membrane-associated guanylate kinase (MAGUK) protein family of zonula occludens (ZO1, ZO2, and ZO3) (Fig. 1) [12]. Another set of junction proteins, the adherens, are involved in the development, stabilization and organization of the intercellular junctions at the endothelium, and involve cadherins, catenins, PECAM-1, and the junctional adhesion molecules (JAMs) -A, -B, -C and endothelial cell-selective adhesion molecule (ESAM) [12, 13].

The claudins, specifically claudin-5, are considered the primary sealing component of the tight junctions [10, 11]. Claudin-5 and occludin are both transmembrane proteins, while ZO-1 is a peripheral membrane protein. Claudin-5 contributes to the reduced paracellular ion movement and helps narrow the paracellular cleft [11, 14, 15]. The functionality of claudin-5 may also rely on Rho-associated protein kinase signaling and phosphorylation of the claudin-5 [16]. Occludin is also present in the filaments of tight junctions and helps regulate adhesion properties between cells as well as interacting with the inner cellular scaffolding proteins and the actin cytoskeleton [11]. ZOs are peripherally associated proteins that interact with claudins, occludins, and JAMs to anchor the membrane proteins, tethering them to the actin cytoskeleton [10, 16].

### BBB transporters

**Fig. 2:**
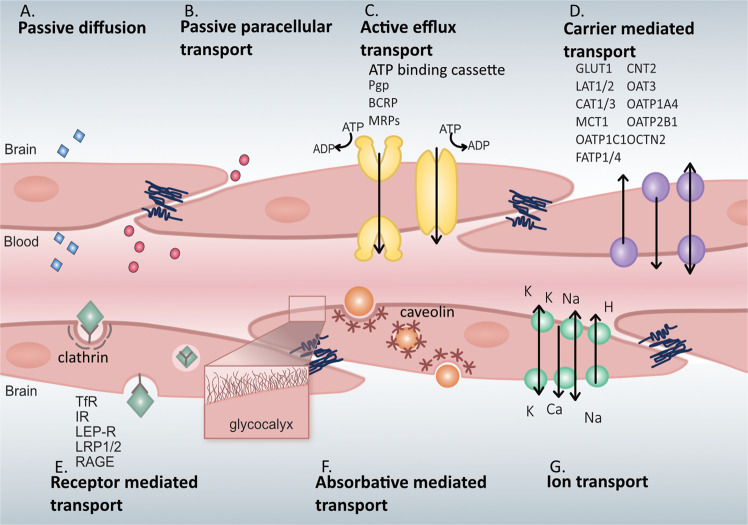
Transport mechanisms across the blood-brain barrier. **A** Passive diffusion across endothelial cells by a limited number of small molecules (blue). **B** Paracellular transport of limited water-soluble agents (pink) between endothelial cells, through tight junction proteins. **C** Active efflux transporters (yellow) mostly eliminate drugs and substanes from the brain include many ATP binding cassette (ABC) transporters (purple) which are P-glycoprotein (Pgp), multidrug resistance proteins (MRPs), and breast cancer resistance protein (BCRP). **D** Carrier-mediated transport can be in either direction depending on the transporter and can be clatherin-dependent endocytosis. Major transporters include the glucose carrier (GLUT1), the L-type amino acid transporter 1 and 2 (LAT1/2), cationic amino acid transporter 1 and 3 (CAT1/3), the monocarboxylic acid carrier (MCT1/8), the organic anion transporting polypeptide 1c1 (OATP1C1), the fatty acid transport protein 1 and 4 (FATP1/4), the sodium-independent concentrative nucleoside transporter-2 (CNT2), the organic anion transporter 3 (OAT3), organic anion transporter poypeptide 1a4 and 2b1 (OATP1A4 and OATP2B1), and the organic cation transporter 2 (OCTN2). **E** Receptor-mediated transport relies on the interaction between ligands (green) and receptors to transport larger molecules through the cells. These receptors include the transferrin receptor (TfR), insulin receptor (IR), leptin receptor (LEP-R), lipoprotein receptor 1 and 2 (LRP1/2), and the receptor for advanced glycation end products (RAGE). **F** Absorptive-mediated transport is caveolin-mediated endocytosis and relies on the interaction between the ligand (orange) and the glycocalyx on the endothelial cells. **G** Ion transporters (turquoise) regulate the ions between the barrier and includes sodium pumps, calcium transporters, and potassium channels.

There are a limited number of solutes that can cross the BBB without the use of transporters. The only molecules that can passively diffuse across the BBB are gases such as oxygen and carbon dioxide, and small lipid-soluble molecules with a molecular weight under 400 Da or containing less than 8 hydrogen bonds (e.g., ethanol, antidepressants) [17]. Passive paracellular transport of water-soluble agents is very limited in the BBB due to the presence of the tight junctions. To accommodate all the other components necessary to keep brain homeostasis, the BBB is equipped with a range of different transporters to ensure that essential molecules can readily enter the brain. Beyond passive diffusion and transcellular transport, these transporters can be broken down into five additional categories: active efflux transport, carrier-mediated transport, receptor-mediated transport, absorptive-mediated transport, and ion transporters (Fig. 2).

Active efflux transport (Fig. 2C) largely encompasses the ATP binding cassette (ABC) class of transporters. ABC transporters require energy in the form of ATP to transport molecules across the concentration gradient. These transporters largely prevent the accumulation of drugs, drug conjugates, nucleosides, and xenobiotics in the brain [12]. Examples of these transporters include P-glycoprotein (Pgp), multidrug resistant proteins (MRPs), and breast cancer resistant protein (BCRP). ABC transporters are largely known to actively prevent accumulation of drugs and other agents in the brain [12, 18, 19].

Carrier-mediated transporters (Fig. 2D) are highly selective and generally facilitates the transport of nutrients from the blood to the brain although they can be bi-directional depending on the concentration gradient [20, 21]. Many of these transporters also belong to the superfamily of solute carriers (SLC). Examples of some of the SLC transporters present on brain endothelial cells include organic anion transporting polypeptides (OATPS) and organic cation transporters (OCTS). Carrier-mediated transporters are responsible for transporting several molecules such as carbohydrates, amino acids, monocarboxylates, hormones, fatty acids, nucleotides, organic anions, and cations etc. Some examples of the carrier-mediated transporters include the glucose carrier 1 (GLUT1), the LAT1/2, cationic amino acid transporter 1 and 3 (CAT1/3), the monocarboxylic acid carrier 1 and 8 (MCT1/8), the fatty acid transport protein 1 and 4 (FATP1/4), the sodium-independent concentrative nucleoside transporter-2 (CNT2), the organic cation transporter 2 (OCTN2), the OAT3, and the organic anion transporting peptides (OATP1C1, OATP1A4, OATP2B1) [12].

Receptor-mediated transport (Fig. 2E) requires the binding of a ligand to a receptor on the plasma membrane and is responsible for the transport of proteins and peptides both into and out of the brain [22, 23]. Examples of some of the receptors involved in receptor-mediated transport include the transferrin receptor (TfR), insulin receptor (IR), leptin receptor (LEP-R), lipoprotein receptor 1 and 2 (LRP1/2), and the receptor for advanced glycation end products (RAGE). Both the TfR and IR have been widely utilized to transport CNS targeting drugs across the BBB via the trojan horse strategy [24]. Many of these receptors are clathrin-dependent endocytosed, one example being LRP1 which is responsible for the endocytosis of amyloid-beta and APOE 2 and 3 [12].

The other major form of endocytic pathway in brain endothelial cells is caveolae-dependent. In this case molecules are absorbed in the absence of receptors, but rather through charged interaction between the ligand and the glycocalyx of endothelial cells and can be referred to as absorptive-mediated transcytosis (Fig. 2F) [18]. Although controversial, albumin may transcytose through the BBB via caveolae-mediated vesicular trafficking, which although present, is greatly restricted in the BBB [18]. Another form of vesicular transport pinocytosis, which is responsible for the transport of fluids across cells, is largely lacking in the BBB [25]. Brain endothelium is also equipped with ion transporters (Fig. 2G) such as sodium pumps, calcium transporters, and potassium channels. These are critical to regulate the electrophysiological activity of neuronal cells, to maintain the sodium concentration gradient at the BBB that drives sodium-dependent transport processes, and to regulate the intracellular endothelial pH [12].

### Pericytes

Pericytes are vascular mural cells that grow in close proximity to the brain endothelial cells, embedded in the basement membrane, covering ~90% of the capillary bed [26]. Vascular mural cells also include vascular smooth muscle cells (vSMC) which surround large vessels and both cell types support blood vessels. Importantly, pericytes constitute a heterogeneous cell population, and the role of each sub-class in NVU function is currently unknown. Distinguishing pericytes from vSMC is particularly challenging as they seem to have a shared, albeit heterogeneous origin [27], which leads to a continuous gradient of sub-types between canonical vSMC and canonical pericytes, with features of both cell types. Current identification and classification of different sub-types of mural cells is based on a combination of antigen markers, spatial distribution, and morphological features. For the latter, high resolution imaging techniques have allowed to identify three main pericyte morphologies: ensheathing pericytes (mostly associated to capillary and post-capillary venules), mesh pericytes (mostly associated arteriole-capillary junctions), and thin strand pericytes (mostly associated to the middle of capillaries) [28].

Both endothelial cells and pericytes contribute to generate components of the basement membrane, which is a vital component of the BBB, providing structural support, cell anchoring, regulate immune cells, and signaling transduction [1, 29, 30]. The four major proteins making up the basement membrane are collagen IV, laminin, nidogen, and perlecan [30]. Microvascular pericytes are particularly important as they contribute to maintenance of the BBB, regulate cerebral blood flow, play a key role in generation of new blood vessels, aid microvessel stability, and clear waste from the brain [31].

Endothelial cells directly communicate with pericytes by secreting platelet-derived growth factor B (PDGF-B) which binds to its receptor expressed by pericytes, PDGF receptor beta (PDGFRβ) [32]. This binding activates signal transduction pathways involved in regulating proliferation, migration, and recruitment of pericytes [33]. These signals are particularly important during angiogenesis when pericytes are recruited in response to the PDGF-BB secreted from endothelial cells [31]. The adhesion between endothelial cells and pericytes is mediated by transforming growth factor-β (TGF-β) which can be secreted by both cell types. Both cell types can also respond to TGF-β through TGF-β receptor. Pericyte TGF-B signaling supports the BBB integrity by promoting fibronectin expression, basal membrane synthesis, and stimulating tight junction protein expression [34].

Pericytes themselves help maintain BBB integrity and decrease paracellular permeability of endothelial cells by enhancing the formation of tight junctions and maintaining low levels of transcytosis in the brain endothelium [35–37]. Pericytes also regulate the gene expression patterns of BBB-specific patterns in endothelial cells and induce polarization of astrocyte end feet [38, 39]. Furthermore, in mouse models with defects in pericyte generation, there is a failure to downregulate genes associated with increased endothelial permeability such as angiopoietin (Angpt2) and plasmalemma vesicle-associated protein (Plvap) gene [35, 40].

The extravasation and trafficking of leukocytes in and out of the brain are also influenced by factors that can be released from pericytes and endothelial cells [31]. Pericytes also react to cytokines and triggers the release of proinflammatory molecules, which leads to BBB breakdown in vitro and induces activation of a proinflammatory state of astrocytes, endothelial cells, and microglia, the brains innate immune cell [31]. Importantly, pericytes play an important role in cerebrovascular malfunction in Alzheimer’s disease (AD) [41]. In this context, carriers of apolipoprotein 4 (APOE4) allele, a major risk factor for AD, present with pericyte degeneration and associated BBB breakdown [42], which has been recently associated with cognitive decline, independent of AD pathology [43].

### Astrocytes

Astrocytes, a type of glia cell, play a major role in maintaining the integrity of the BBB. This is exemplified in animal models which temporarily reduce astrocyte expression and have decreased expression of tight junction proteins, which is restored to normal levels when astrocytes are repopulated into the targeted region [44]. Astrocytes can affect the expression and polarized localization of transporters including Pgp and GLUT1 [45]. Although the mechanisms through which astrocytes maintain BBB integrity are not fully understood, there are several proposed astrocytic-derived soluble factors that may induce vital aspects of the BBB including interleukin-6 (IL-6), glial cell line-derived neurotrophic factor (GDNF), basic fibroblast growth factor (bFGF), fibroblast growth factor 2 (FGF-2), angiopoetin 1 (ANG1), and TGF β [45, 46]. Astrocytes may be involved in the maintenance of BBB integrity and help restore a vessel when leaky since implantation of cultured astrocytes into normally leaky vessels results in tightening of the endothelium [45]. Astrocytes also have a distinct role in contributing brain immunosurveillance, having the capability to present antigens and produce cytokines and may affect BBB permeability to allow entry of circulating T cells into the brain [47].

An important feature of astrocytic interaction with the BBB are the perivascular end feet which closely interact with the vascular walls and almost completely cover the entire cerebral vasculature [48]. These end feet have a high density of orthogonal arrays of particles which contain the water channel aquaporin 4 (AQP4) and the potassium channel, Kir4.1, which are involved in ion and water regulation [45]. Astrocytic end feet do not significantly contribute to the physical barrier in a direct way. However, they play a key role in the formation and maintenance of functional BBB properties [18]. Moreover, they also secrete extracellular matrix proteins, laminins, that are part of the unique basement membrane in the NVU and also contributes to the BBB [49]. Astrocytic endfeet appear swollen under certain pathological conditions such as ischemic stroke [50], which has been associated with changes in osmotic homeostasis in response to the release of factors such as L-glutamate of K^+^ from parenchymal brain cells.

## BBB disruption in health and disease

**Fig. 3:**
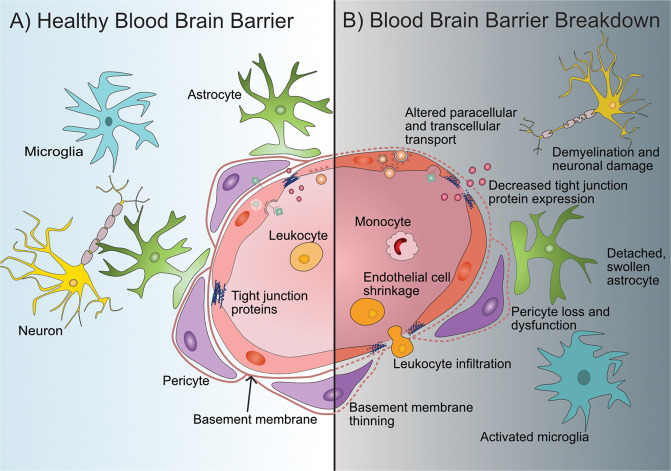
Schematic representation of the blood-brain barrier (BBB) in a healthy state and during BBB breakdown. **A** Healthy, intact BBB structure and surrounding cells and key components. Endothelial cells form the main physical barrier lining the blood vessels in the brain with tight junction proteins between them. Leukocytes are in constant circulation. Endothelial cells are encompassed by the basement membrane which also encompasses pericytes which are in close contact to the endothelial cells. Astrocytic endfeet interact closely with the endothelial cells and pericytes and help maintain BBB integrity. Inactivated microglia and functional neurons are present in a healthy neurovascular unit. **B** During BBB breakdown its integrity can become compromised at various levels. Disruption characteristics of the BBB include endothelial cell alterations such as loss of tight junction proteins, endothelial cell shrinkage, changes in molecular transport at the paracellular level, and transcellular level in some cases, and increased leukocyte infiltration. In some disruption models pericyte dysfunction or loss is apparent as well as astrocyte changes such as swollen or detached endfeet. Microglia can also become activated and neurons may experience demyelination or become damaged.

Nearly all aspects of the BBB can be altered in BBB breakdown when compared to a healthy BBB (Fig. 3). The alterations and breakdown of functional components of the BBB can occur naturally with aging even in the absence of underlying conditions that cause cognitive decline and dementia [51]. The disruption that occurs in healthy aging may become more detrimental when exposed to a second hit, such as inflammation. Notably, the BBB may be disrupted in aged mice, but cognitive decline is not apparent until there is an inflammation challenge [52]. The morphological and molecular alterations of the BBB happen in the absence of disease pathologies. Healthy aging is defined as the accumulation of time dependent cellular damage which factors include oxidative stress, epigenetic changes, genomic instability, telomere attrition, and dysregulation of cell signaling and inflammatory responses, upon further hit, this natural disruption may become detrimental [53].

BBB breakdown often involves endothelial cell degradation or shrinkage [12, 46] and altered paracellular transport pathways via decreased expression of tight junction proteins and/or tight junction translocation [51]. Furthermore, expression or function of BBB-associated receptors and transport mechanisms can be affected leading to dysregulated molecular transport [12, 54]. Importantly, BBB breakdown can also involve different cellular elements beyond brain endothelial cells. These include pericyte degeneration or reduction in pericyte coverage [54, 55], basement membrane alterations [56], and astrocytic end-feet detachment from vascular basement membrane. These astrocytic end-feet may also present a swollen phenotype under certain pathological conditions [46, 57].

Although not considered a change in structure to the BBB per se, endothelial cells can increase leukocyte adhesion molecule expression leading to increased leukocyte extravasation into the brain parenchyma under pathological conditions [58, 59]. Moreover, these leukocyte-endothelial cell interactions can also directly contribute to increase BBB permeability by releasing reactive oxygen species, cytokines, and other mediators of barrier disruption [60].

Importantly, BBB can be also endogenously repaired, but we have limited knowledge yet on what mechanisms and which cellular components of the NVU are involved in regulating BBB repair. In this context, astrocytes and microglia have been shown to play a role in repairing the BBB in response to injury [60]. Microglia seem to play an important and dynamic role in BBB integrity in response to different insults. Resident microglia are considered part of the NVU due to their capacity to modulate BBB function in response to changes associated with various conditions. Microglia interact directly with brain endothelial cells [61] and have been shown to play dual roles in BBB modulation upon different insults. Upon stroke, microglia initially respond by contributing to BBB disruption via secretion of proinflammatory cytokines, but at later stages microglia contribute to BBB repair [61]. Interestingly, systemic inflammation has a somewhat opposite effect on microglia-BBB interactions, as initially microglia respond to systemic inflammation by maintaining BBB integrity through direct interactions with endothelial cells. However, sustained inflammation promotes BBB disruption by inducing microglial-led phagocytosis of astrocytic end-feet [62].

### Key driving factors of BBB disruption

Inflammation induces a series of changes in BBB function and physiology. Under inflammation, glia cells become activated, leukocytes are recruited across the BBB, and the BBB itself breaks down, with alterations in paracellular and transcellular transport and tight junction protein expression [63–67]. Several models of inflammation are used to better understand these effects including viral RNA, systemic infection, non-infective systemic inflammation, inflammatory mediators, and cytokines, but the most widely accepted model is lipopolysaccharide (LPS) challenge. LPS is a bacterial endotoxin which promotes secretion of proinflammatory cytokines and activates the immune response [68–70]. In in vitro models, LPS disrupts the barrier with decreased TEER and increased permeability [67]. In agreement with the decrease in TEER and increase in BBB paracellular permeability, tight junction proteins and adherens junction protein, β-catenin, expression and localization are also altered with LPS treatment [71]. Endothelial apoptosis, membrane abnormalities, and mitochondrial damage are all features of systemic inflammation induced by BBB disruption [67, 71]. Inflammation also alters transcellular transport mechanisms. Active efflux transporter, PgP, solute carrier transporters, OATs, and carrier-mediated transporters, MCT8 and LAT1, and the receptor-mediated transporter, LRP-1, decrease in activity, mRNA expression, or protein expression are also reduced following LPS challenge [67, 72–75].

With systemic inflammation, both astrocyte and pericyte interactions with endothelial cells alter. There is a downregulation of proteins involved in pericyte-endothelial cell communication [76–78] and astrocytes, being a glia cell, become activated. In some cases, astrocytes experience structural changes such as swollen end feet. The consequences and cause of the swelling are or not fully understood yet, but some hypothesize a swollen end foot may help cuff a damaged vessel [79]. This is however controversial and astrocytic morphology changes are not always seen [67, 80].

Oxidative stress disrupts the integrity of the BBB through mechanisms that result from an excess of reactive oxygen species (ROS) accompanied by a compromised intrinsic antioxidant defense [81]. Oxidative stress occurs when the oxidant-antioxidant balance is disrupted that leads to excess oxidants [81]. Examples of ROS include superoxide, hydrogen peroxide, peroxynitrite, nitric oxide, and hydroxyl radicals. Nitric oxide is the primary oxidative species contributing to BBB damage. The high reactive oxygen consumption by the brain enhances the brain’s susceptibility to oxidative stress to which endothelial cells are more sensitive compared to pericytes, and pericytes are more sensitive than astrocytes [82]. Cell culture models of oxidative stress include glucose deprivation, hypoxia or hydrogen peroxide treatment [83, 84]. Animal models can include exogenous treatment with free radicals, stroke, or transgenic mouse models which uses normal metabolic processes to produce a pro-oxidative state [85]. The disruption mechanisms of oxidative stress include oxidative damage to cellular components (protein oxidation, lipid peroxidation, and DNA damage), activation of matrix metalloproteinases, cytoskeleton reorganization, modulation of tight junction proteins, and upregulation of inflammatory mediators [81, 86, 87]. The disruption compromises the structural integrity affecting the cells capacity for cell transport, energy production, and ion balance [87]. From within cells, ROS can disrupt pathways including the small GTPase RhoA, PI3, and protein kinase B (PKB/Akt) signaling pathways. This disruption leads to a rearrangement of actin cytoskeleton and altered localization of occludin and claudin-5 [88]. There is further evidence that hypoxic stress is correlated with ZO-1 localization and BBB permeability. Because occludin, ZO-1, ZO-2, and claudin-5 are phosphoproteins, any changes in phosphorylation may result in altered BBB permeability [88].

### BBB disruption in healthy aging

The BBB undergoes a number of deleterious changes during normal aging. Some of these changes may be adaptive but probably also contribute to age-associated cognitive decline and diseases. This topic has been recently reviewed extensively elsewhere [53]. During healthy aging, there is an age-dependent loss of BBB integrity in the hippocampus of individuals who have no cognitive impairment as seen with magnetic resonance imaging (MRI) [89]. In clinical settings, MRIs are useful when determining BBB permeability through acquisition of images during the passage of a contrast agent. Aged mice also have hallmarks of BBB breakdown compared to young mice with increased leakage of IgG into the brain parenchyma, a reduction of the tight junction protein occludin, and a reduction in pericyte coverage and pericyte induced expression of genes in the endothelium [54, 90, 91]. The uptake of glucose into the brain is also reduced in aged healthy individuals as well as aged rodents indicating there may be a change in expression or function of the GLUT1 transporter with age [91–94]. Additionally, LRP-1 is decreased with age in mice [95] and Pgp function decreased with age in both humans and mouse models [91, 96–98]. Changes in BBB transport are also evident by a decrease in cerebralspinal fluid to serum ratio of insulin [91, 99]. There is an age-related shift in transport from receptor-mediated transport to caveolar transcytosis which may account for some of these changes mentioned, namely an increase in IgG antibody, but a decrease in plasma protein uptake [54]. Interestingly, systems regulating the circulation of brain extracellular fluid and cerebrospinal fluid, such as the glymphatic system, are also decreased with healthy aging [53, 100].

### BBB disruption in age and neurodegenerative disorders

Disruption of the BBB coincides with healthy aging, but the characteristics of breakdown can become further exacerbated in neurodegenerative disorders (Table 1). BBB disruption is a hallmark associated with several pathologies including the neurodegenerative diseases and is often found to a greater extent in disease compared to healthy aging. BBB breakdown is well characterized in AD, Parkinson’s disease (PD), Amyotrophic lateral sclerosis (ALS), Multiple Sclerosis (MS), and Huntington’s disease (HD).

**Table 1:** Diseases in which blood-brain barrier disruption is a hallmark of this disorder and the subsequent involvement of oxidative stress and inflammation.

Disorder	BBB dysfunction	Inflammation and oxidative stress	References
Alzheimer’s Disease	Increased paracellular permeability (MRI, postmortem analysis, increased albumin CSF ratio, TEER, fluorescein). Decreased tight junction proteins claudin-5, occludin, and ZO-1 expression. Degradation of endothelial cells and pericytes. Alterations in thickness of the basement membrane. Astrocyte degeneration. Decreased expression of GLUT1 transporter and decreased levels of low density-lipoprotein receptor-related protein (LRP). Increased expression of receptor for advanced glycan end products (RAGE).	Increased pro-inflammatory cytokines IL-1β, IL-8, TNF. Activated astrocytes. Activated microglia. Leukocyte infiltration. Increased ROS.	[55, 56, 95, 101, 105, 112, 160–169]
Parkinson’s Disease	Increased permeability (Albumin leakage, increase hemoglobin, fibrinogen, 70 kDa FITC, TEER). Decrease in expression of tight junction proteins claudin-5, occludin and ZO-1. Decreased endothelial cells and cell thickness. Increased thickness of the basement membrane. Activation of pericytes. Altered activity and expression of Pgp efflux transporter. Decreased GLUT1 and BCRP expression.	Increased proinflammatory cytokines IL-1 β, IL-2, IL-6, TNF Activated astrocytes. Activated microglia. Leukocyte infiltration. Increased ROS.	[12, 102, 103, 170–175]
Amyotrophic lateral sclerosis	Increased barrier permeability (hemoglobin, increased albumin, Evan’s blue leakage). Decreased abundance of claudin-5, occludin, and ZO-1. Endothelial cell degeneration or damage. Basement membrane changes in PECAM-1 and collagen IV proteins. Altered astrocyte phenotype, astrocyte end-feet capillary damage and dissociation. Pericyte degeneration or damage.	Increased proinflammatory cytokines IL-1β, IL-2, IL-6, IL-10, TNF. Activated astrocytes. Activated microglia. Leukocyte infiltration. Increased ROS.	[176–187]
Multiple Sclerosis	Increased barrier permeability (MRI, Evan’s blue). Reduced expression of tight junction proteins claudin-5, occludin, and ZO-1. Reorganization of actin cytoskeleton. Changes in pericyte morphology and swollen astrocytic end feet.	Increased proinflammatory cytokines IL-2, IL-17, TNF. Activated astrocytes. Activated microglia. Leukocyte infiltration. Increased ROS.	[188–196]
Huntington’s Disease	Increased barrier permeability (MRI, postmortem analysis, increased albumin). Decreased expression of claudin-5 and occludin. Altered pericyte coverage and density. Altered transcellular vesicular transport. Decreased GLUT1 expression on astrocytes. Reduction of pericyte coverage.	Increased proinflammatory cytokines IL-1β, IL-6, IL-8, TNF. Activated astrocytes. Activated microglia. Increased ROS.	[197–206]
Ischemic Stroke	Increased barrier permeability (sucrose leakage, Evan’s blue, MRI). Decreased expression of tight junction proteins claudin-5, occludin, and ZO-1. Degraded basement membrane. Swollen astrocytic end-feet. Increased GLUT1 expression. Alterations in solute carrier expression. Increased vesicular transport.	Increased proinflammatory cytokines IL-1β, IL-6, TNF. Activated astrocytes. Activated microglia. Leukocyte infiltration. Increased ROS.	[9, 46, 50, 207–212]

In many neurodegenerative diseases, the exact cause and pathology remains unknown, making it difficult to know whether the BBB dysfunction in the disease is a causative agent, a result of the disease, or somewhere in the middle. An increase in BBB permeability is commonly observed via enhanced accumulation of blood-derived proteins in the brains of post-mortem AD patients [101] and supported by the reduction of tight junction proteins [102]. Further changes in BBB integrity have been noted with changes in expression and function of BBB transporters and receptors such as GLUT1, LRP, or Pgp [103–111]. In addition to alterations in the mechanistic functions of brain endothelial cells, BBB breakdown is evident by degeneration of both endothelial cells and pericytes [101]. Oxidative stress and inflammatory dysfunction of the BBB is evident by ROS activity and an increase in inflammatory mediators [112].

## Restoration of BBB integrity

The disruption of the BBB in the neurodegenerative disorders makes it a clear target for therapeutics. If the BBB disruption could be restored, at least to some extent, it may be possible to slow the pathogenesis of the disease. Tightening the barrier could reduce the negative effects of the inflammation challenge or in the presence of any of the neurodegenerative diseases, controlling the integrity of the BBB could slow the progression of the disease and neurodegeneration. Before diving into the two distinct categories of therapeutic restoration of BBB integrity, drug therapy and microbial metabolites, it is first necessary to introduce the tools used for studying the BBB, to better understand how the therapeutics are screened for restoring barrier integrity.

### Tools to study BBB disruption and restoration: focus on in vitro models

**Fig. 4:**
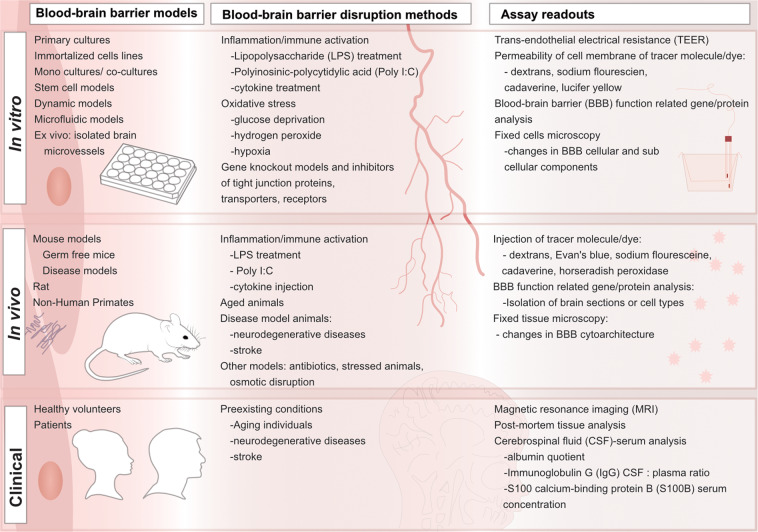
Models for studying the blood-brain barrier in health and disease with the respective disruption methods simulating disease and readouts. In vitro, in vivo, and clinical models have all been used to study blood-brain barrier (BBB) integrity in both health and disease models. Depending on the model of interest, different methods are used to mimic BBB loss of integrity. The assays available to observe and quantify are also different depending on the model of interest. These models, disruption methods, and readouts are highlighted here.

The three major categories of BBB models include in vitro systems, in vivo models, and clinical models (Fig. 4). Clinical models are useful for understanding how the BBB is functioning and disrupted in patients with disease, however drugs and therapeutics are not screened in clinical models until there is some certainty using in vivo and models that the therapeutic is safe and effective. In vivo models are more accessible than clinical models and can be used to screen a few hit therapeutics to determine safety and efficacy in whole system living organisms. The most accessible models are models that can readily be used to screen several therapeutics at once before moving in to living organisms. A summary of the BBB models, disruption methods associated with each model and assay readouts are highlighted in Fig. 4. Since many of the proposed therapeutics are in early days of development there is a particular focus on the importance of in vitro systems in screening novel therapeutics.

In vitro BBB models are widely used to study BBB physiology and the cellular mechanisms involved in this barrier as it can be readily manipulated. The simplest of these in vitro models is a monoculture of brain endothelial cells on transwell membrane inserts or standard flat tissue culture plates. Transwell inserts allow the endothelial cells to grow on a membrane that sits in a stagnant tissue culture plate and is useful to measure the barrier properties of the subsequent monolayer. Co-cultures of brain endothelial cells with astrocytes, pericytes, neurons, or microglia or some combinations have also been widely explored using transwell inserts. The co-cultures are useful as the presence of either astrocytes or pericytes or both cells together with brain endothelial cells improves the BBB characteristics [36, 38, 39, 113, 114]. Adding neurons to the culture makes the model more representative of the interaction in the NVU and increases the sensitivity of the BBB integrity to oxygen-glucose deprivation [115]. This also highlights the role of neurons in contributing to BBB maintenance and breakdown [115]. Other models use a co-culture of astrocytes, pericytes, neural stem cells and human induced pluripotent stem cell-derived BBB endothelial cells to improve BBB integrity [116].

In co-cultures, endothelial cells are grown on the basolateral side of the transwell inset on the membrane, while the other cell types are grown on the underside of the membrane or in the base of the cell culture dish. Because of the simplicity of traditional cell culture plates or transwell filters, they are useful for screening multiple compounds and looking at many mechanistic outputs, while the other models are often used for more specialized readouts. Other more complex models include dynamic BBB models and microfluidic models/3D chip-style models. The dynamic models are designed to incorporate the shear stress that a steady blood flow generates on endothelial cells in normal physiological conditions. The stress of the blood flow helps regulate tight junctions and transporter expression [117]. Examples of these models are the cone-plate apparatus and the dynamic in vitro model. Microfluidic based models also are designed to include the stress of the blood flow in BBB physiology but also closer replicate BBB structure with thicker membranes [117]. These models allow for transmigration studies as well as more precise measurements of TEER making them very useful in permeability studies and reduces the number of cells needed for the model [117].

All the cell culture models can be used with a variety of cell types such as immortalized cell lines, primary cultured cells, and pluripotent stem cells. The simplest and most efficient of these to culture are the cell lines since they can be grown readily and frozen down to be kept and used in the lab for years. These cell lines can be from human, rat, bovine, and mouse origins with hCMEC/D3 and bEnd.3 cells being the most common brain endothelial cell lines [118]. The cell lines, however, lose their BBB characteristics the more they are passaged, decreasing tight junction and transporter expression. Primary cells are another option which have much higher TEER and lower permeability than the available cell lines, but they are more time consuming, expensive, and can be passaged a limited number of times. Stem-cell derived BBB models are the newest cell type model and have significantly higher expression of tight junction protein and transporters as well as TEER compared to hCMEC/D3 cells [118, 119]. The use of stem-cells also offers the unique aspect of being patient specific which is extremely useful in understanding disease pathologies and developing novel therapeutics [117].

In vitro cell models of the BBB offer many advantages such as the ability to test the effects of molecules on specific cell types in a high throughput manner, and to investigate several cellular mechanisms simultaneously and repetitively [120]. Cultured cells offer a way to dive deeper into the mechanism of action behind single entities quicker and more controlled than would be possible in animal or clinical models.

### Drug therapy

Some drugs have been explored for their therapeutic effect, tightening the BBB primarily in cases of ischemic stroke, but several therapeutics have also showed promise in the context of the neurodegenerative diseases MS, ALS, and AD.

There are two waves of BBB disruption in ischemic stroke. The first wave of disruption is caused by a sudden loss of blood flow to the brain which can quickly lead to metabolic disturbances, inflammation, oxidative stress, and neuronal death, while the second wave occurs at the reperfusion of oxygenated blood into the affected area, putting oxidative stress on the system. For these reasons, several drugs have been explored for their protective effects in oxidative stress in vitro models and ischemic stroke mouse models (middle cerebral artery occlusion/reperfusion). One drug, 10-O-(N, N-dimethylaminoethyl) ginkgolide B methanesulfonate (XQ-1H) has previously been explored for its involvement in pharmacological activities including antagonizing platelet activating factor, suppressing oxidative stress and neutrophil infiltration [121–123]. XQ-1H protects against oxygen and glucose deprivation/reoxygenation in vitro with increased TEER, increased cell viability, increased tight junction protein expression, and decreased permeability [122]. XQ-1H alleviates BBB leakage in ischemic stroke model mice increasing the expression of claudin-5, occludin, ZO-1, and B-catenin [122]. XQ-1H also protects BBB integrity in rats and further protects against LPS induced inflammatory response in brain microvessel endothelial cells [121]. Anther drug, JLX001, the dihydrochloride of cyclovirobuxine D (CVB-D) also has protective effects over the BBB. In primary cultured microvascular endothelial cells and in rats, JLX001 protects against glucose deprivation and reoxygenation [124]. JLX001 increases the expression of the tight junction proteins, claudin-5 and occludin, through activating Wnt/B-catenin signaling pathway [124]. Exosomes harvested from bone marrow stromal cells obtained from type 2 diabetic rats have also been explored as a potential therapy following stroke. These exosomes improve neurological function as well as decrease BBB leakage by decreasing leakage in hemorrhage and increasing tight junction protein ZO-1 expression [125]. Another drug, activated protein C, reduces BBB breakdown and leakage in stroke model rodents and has anti-inflammatory effects, neuroprotective effects, and blocks hemorrhage after brain ischemia [126, 127]. Activated protein C which protects the BBB integrity also has therapeutic effects in MS, ALS, and AD [128–130]. This is a protease that has anticoagulant activity and inhibits BBB breakdown, neuronal damage, and inflammatory responses [131].

MS is an autoimmune and inflammatory neurological disease of the CNS that damages the myelin sheath surrounding and protecting nerve cells [132]. The mechanisms of the BBB help regulate the immune responses of the brain and controls the exchange of immune cells between the blood and the brain. Activated protein C however has not been explored for it’s protection over BBB integrity in the context of MS, but rather for it’s anti coagulation effects, reducing disease severity in MS models [128]. The aspect of BBB protection from this molecule warrants investigation in the context of MS as another pathway in slowing disease progression. Additionally, patients with MS show a selective downregulation of the protein annexin A1 in the plasma and cerebral microvessel endothelia and annexin A1 knockout mice have increased BBB permeability [133]. The anti-inflammatory protein, recombinant annexin A1, therefore makes for a great candidate therapeutic. This anti-inflammatory protein also decreases BBB permeability and restores integrity in endothelial cells through cytoskeleton interactions in cultured brain endothelial cells [133].

Amyotrophic lateral sclerosis (ALS) is a chronic neurodegenerative disorder effecting nerve cells in the brain and spinal cord causing loss of muscle control [134]. One of the genetic factors to have a link to ALS is sporadic mutations in the antioxidant enzyme Cu/Zn superoxidase dismutase 1 (SOD1) [135]. Activated protein C downregulates SOD1 in SOD1 mutant mice, reduces blood-spinal cord barrier permeability, and slows disease progression [130]. Another aspect of slowing disease progression of ALS could be through protection of the BBB, but this warrants further investigation.

AD is a neurodegenerative disorder with two main pathological hallmarks of amyloid-beta plaque buildup and formation of neurofibrillary tangles [102]. As summarized above, there is a breakdown of the BBB in AD along with an increase in oxidative stress and inflammatory disfunction [112]. Previous therapeutics that target the amyloid cascade pathway have failed to alleviate AD pathology and restore cognition and memory, therefor new approaches are needed to slow or prevent AD. Since BBB breakdown and vascular dysfunction are a hallmark of AD, therapeutics targeting the BBB are of great potential [136]. Similar to the protease discussed in the context of stroke, MS, and ALS, the cell-signaling analog of activate protein C, 3K3A-activated protein C, in addition to improving cerebrovascular integrity also diminished neuroinflammatory responses and slows the generation of amyloid-beta plaque buildup in AD model mice [129]. This emphasizes the diverse effect of activated protein C on slowing disease progression in neurodegenerative disorders and improving BBB integrity in several disease models. Other potential therapeutics include specific inhibitors and genetic manipulation of cyclophilin A (CypA) which can ameliorate the vascular and neuronal dysfunction found in AD model mice through inhibition of the CypA — nuclear factor kappa B (NF-κB) — matrix metalloproteinases-9 pathway [137, 138]. NF-κB is a family of transcription factors involved in regulation of the inflammation and matrix metalloproteinases are activated under oxidative stress. AD patients have increased glutamate and one of the ways the BBB is disrupted in AD patients is with reduced GLUT1 expression, but the GLUT1 stimulator ceftriaxone has been shown to improve hippocampal memory and synaptic plasticity impairment in AD model mice [138, 139]. Another potential therapeutic, Minocycline is a microglial inhibitor which reduces BBB dysfunction by preventing production of glutamate, matrix metalloproteinases, and the proinflammatory cytokine, IL-1β, and increasing the levels of cells responsible for the remyelination of neurons which would be relevant in treating AD [138, 140–142]. By reducing the production of these, glutamate levels lower, the consequences of oxidative stress are reduced, and pro inflammatory cytokines are reduced, reducing the effects of inflammation and oxidative stress in progressing the disease and disruption of the BBB. Another drug explored as an AD therapeutic is Axitinib, which is a small molecule tyrosine kinase inhibitor that targets vascular endothelial grown factor receptors and is used as an anticancer drug. Axitinib decreases the disruption of tight junction proteins and reduces permeability of the BBB in AD disease mice while also increasing spatial awareness, exploration, associative memory, working memory and lowered amyloid-beta, indicating the potential for this drug to slow disease pathology [136]. The drugs that act on BBB integrity in the context of neurodegenerative disease warrant further investigation to understand the impact of restoring BBB integrity and protecting against breakdown.

### Microbial metabolites

In contrast to drug therapy, recently, gut-derived microbial metabolites have been explored for their potential in modulating the BBB. Interestingly, changes in the gut microbiota have been associated with changes in the brain and pathologies of conditions such as the neurodegenerative disorders [143]. This connection between the gut microbiota and the brain is referred to as the gut-brain axis, a field which has attracted increasing levels of interest over the past two decades. Some of the major influencers of the gut microbiota composition, and therefore brain and behavior, are diet, exercise, environment, age, drugs and medications, and infections [143]. Furthermore, components of the foods we ingest are metabolized by the microbes residing in our guts producing metabolites important for our health [144]. There are many categories of distinct microbial metabolites including short chain fatty acids (SCFAs), bile acids, neurotransmitters, and other bioactive molecules of microbial origin produced in the gut which influence brain signaling. Very few have been explored for direct interactions with the BBB thus many opportunities still exist to explore the physiological effects of these metabolites [145, 146].

SCFAs have previously been implicated in processes such as gastrointestinal function, blood-pressure regulation, circadian rhythm, and immune function, and more recently explored for the effects on BBB physiology [143]. Specifically, one SCFA, propionate, has protective effects on the integrity of the BBB and protection of tight junction proteins [146]. It is not yet fully understood the mechanisms underlying BBB protection, but studies indicate it may be through a CD14-depenent mechanism, suppressing expression of LRP1, and protection from oxidative stress [146]. Earlier indications that the gut microbiota plays a role in affecting BBB integrity have been shown through the use of LPS from gram negative bacteria. The effects of LPS on BBB integrity have been largely covered in a previous section, since LPS is a major model of systemic inflammation.

The relationship between BBB integrity and the gut microbiota is evident in germ free mice models which lack a microbiota and have increased BBB permeability and altered tight junction protein expression effects, which continue from *in utero* into adulthood [147]. Additionally, rhesus monkeys with altered microbiomes from oral treatment of the antibiotic, amoxicillin-clavulanic acid, have increased BBB permeability to albumin [148]. The antibiotic treatment decreased the relative abundance of Firmicutes, a SCFA producing phylum of bacteria in the gut, which correlates with a decrease in SCFA concentrations and an increase in BBB permeability [148]. Other research has also identified that abundance of Firmicutes plays a role in altering BBB function and found that the ratio of Firmicutes/Bacteroidetes increased in aging mice as well as the alpha diversity (the mean species diversity), while the BBB function became impaired [96]. The aged mice also had compromised learning and memory behaviors and increased anxiety, which suggests that the gut microbiome and the BBB are linked to the deleterious changes in aging brains [96]. In another study mice treated with low-dose penicillin in early life, however, have increased mRNA and protein expression of tight junction proteins in the hippocampus [149]. Antibiotic treatment in mice (which reduced the abundance of Bacteroidetes) also reduced the expression of tight junction protein mRNA expression in the hippocampus, but increased expression in the amygdala [150]. There is not yet a proposed mechanism for how antibiotic treatment affects tight junction expression in specific regions of the brain in these studies, but it could be through changes in metabolite composition or cytokine involvement. It is important to note the regions of the brain that are more susceptible to the changes in BBB integrity as regions like the hippocampus and amygdala are heavily affected in neurodegenerative disease as they are vital for long-term memory and processing emotions and behavior. The differences in these studies may be explained by the different effects the antibiotics have on the gut microbiota composition, species of the models, and timing of the antibiotic treatments. These findings further highlight the modulating effects the gut microbiota composition and specific metabolites like SCFAs, have on the integrity of the BBB.

The SCFA, butyrate, has exhibited protective effects against both PD and stroke. In a mouse model of PD, sodium butyrate increased occludin and ZO-1 protein expression as well as attenuated behavioral impairment and neuronal damage induced by the PD model [151]. Direct injection of sodium butyrate decreased BBB permeability in ischemic stroke model mice and reduced the loss of sensory motor function induced by stroke [152]. Another microbial-derived metabolite, Urolithin A, a coumarin, is found in plasma of healthy adults and derived from ellagitannins found in pomegranates, walnuts, and berries and has both protective effects on BBB integrity pre stroke and therapeutic effects post stroke [153]. Urolithin A treatment has increased hippocampus neurogenesis, decreased reactive gliosis, and reduced inflammation in the middle cerebral artery occlusion mouse model of stroke [153]. The metabolite has also been explored for treatments during healthy aging, AD, and MS [154].

Methylamines are another subset of microbial-derived metabolites that are produced by the microbial metabolism of choline and L-carnitine. Recently, methylamine trimethylamine N-oxide (TMAO) has been explored for its protective effects on BBB function both in vitro and in vivo [155]. The precursor to TMAO, trimethylamine, on the other hand impairs BBB function, emphasizing the need to better understand the relationships between microbial-derived metabolites, host processing of these metabolites and BBB physiology [155]. More recently, another microbial product, p-cresol, a glucuronide, has been found to decrease permeability of the BBB in vitro and has protective effects over LPS induced BBB disruption in mice [156]. The direct effects p-cresol has on BBB physiology may be through functional antagonism of the TLR4 complex, a receptor complex activated by LPS [156]. It is intriguing that SCFAs, coumarins, methylamines, and glucuronides have direct effects on BBB integrity. This emphasizes the profound role a diverse subset of microbial-dependent metabolites have on barrier function; therefore more metabolites must be explored for direct influence on BBB physiology.

Not all components of the gut microbiome, however, are protective over the BBB as seen with LPS and evident with deoxycholic or chenodeoxycholic acid. Deoxycholic or chenodeoxycholic acid are bile acids that can directly interact with the BBB, increasing the permeability and disrupting the tight junction proteins [157]. These bile acids are regulated by the microbiome and concentrations can alter with alteration in the gut microbiota composition. Chenodeoxycholic acid is a primary bile acid synthesized in the liver from cholesterol, which is then stored in the gallbladder before being excreted in the small intestine. Deoxycholic acid, on the other hand, is a secondary bile acid, which means it is formed when primary bile acids undergo microbial mediated transformations [158]. The balance between these beneficial and harmful metabolites depends heavily on the gut microbiota composition, and an altered composition compared to a healthy one can lead to more harm than good. Reducing the interactions between the harmful components of the microbiota and the BBB and increasing the interaction of the beneficial/protective components may help to alleviate some of the detrimental characteristics of a compromised BBB.

**Fig. 5:**
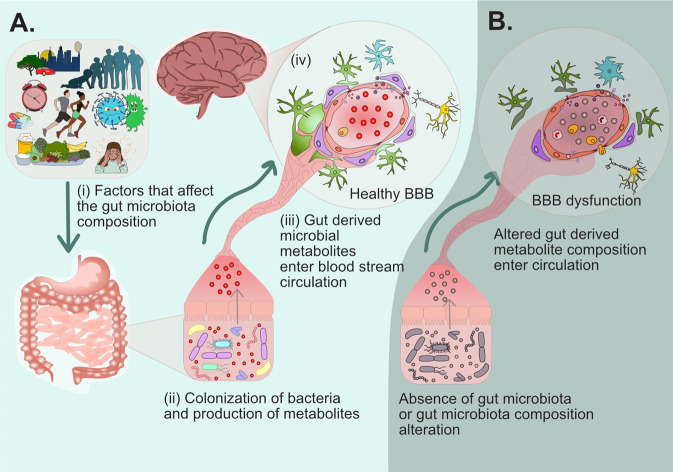
Proposed summary of the relationship between the gut microbiome and the blood-brain barrier. **A** (i) Factors such as the environment, age, circadian rhythm, medication/drugs, exercise, infection, diet and stress can affect the composition and landscape of the gut microbiota. (ii) the gut microbiota and colonized bacteria produce metabolites. These metabolites produced alter with regards to relative concentrations and molecules with changes in microbiota. (iii) gut-derived microbial metabolites cross the gut lumen whether as direct molecules or in derived forms and enter circulation. (iv) once in circulation, the microbial-derived metabolites can interact with the BBB. **B** In the absence of a gut microbiota (germ-free animals) or where there are compositional alterations in the gut microbiota, microbial metabolites are not produced or are differentially produced that can enter systemic circulation and the lack or increase in microbial-derived metabolites is associated with BBB dysfunction.

The interaction between the gut microbes and their metabolites is suggested to introduce a fourth facet of communication to the gut-brain axis [146]. The previously identified and accepted pathways of communication include the nervous system via the vagus nerve, the immune system, and enteroendocrine signaling pathways [159]. The additional pathway would be the direct modulatory effects microbes and metabolites have on the BBB itself, influencing the integrity of the brain’s primary defense mechanism and therefore impacting brain health. This interaction, however, may not be its own individual communication pathway, but rather the BBB is an interface of communication between the gut microbiota, blood, and the brain. The gut microbiota may be interacting with the other pathways such as inflammation, or endocrine which then interact with and affect the integrity of the BBB, but either way it is now apparent that the gut microbiota is a modulator of BBB integrity. In summary, there are several factors that can influence gut microbiota composition which impacts the microbial-derived metabolites that enter circulation, and these metabolites in circulation or lack of metabolites directly interact and influence BBB physiology (Fig. 5). Further understanding of the cellular mechanisms through which microbial metabolites affect BBB physiology would potentially allow microbial-derived metabolites to be exploited for therapeutic protection against BBB breakdown in the context of inflammation, oxidative stress, and age-related diseases.

## Concluding remarks and future perspectives

The BBB function and structure are vital to maintain brain heath and proper function. The disruption of any of the functions of the BBB potentially leads to BBB breakdown or loss of integrity putting brain homeostasis at risk. Deterioration of BBB form and function is a feature part of healthy aging, but it is worsened in many neurodegenerative disorders and is a hallmark of cognitive decline. As the aging population increases, it becomes even more vital to understand the potential mechanisms of future therapies for maintaining and increasing BBB integrity. Emerging research in the gut-brain axis and the protection or disruption by gut microbial-derived metabolites have on BBB integrity are only beginning to be explored. As we begin to better understand the role of the gut-derived metabolites on the system, it will be interesting to incorporate and exploit these interactions for the purpose of therapeutics to either restore or protect against BBB breakdown.
